# Relationship between physical performance and perception of stress and recovery in daily life post COVID-19—An explorative study

**DOI:** 10.1371/journal.pone.0285845

**Published:** 2023-05-15

**Authors:** Jule Zorn, Shirin Vollrath, Lynn Matits, Martin Schönfelder, Sebastian V. W. Schulz, Achim Jerg, Jürgen M. Steinacker, Daniel A. Bizjak

**Affiliations:** 1 Department of Medicine, Division of Sports and Rehabilitation Medicine, Ulm University Hospital, Ulm, Germany; 2 Division of Clinical & Biological Psychology, Institute of Psychology and Education, Ulm University, Ulm, Germany; 3 Department of Sport and Health Science, Division of Exercise Biology, Technical University Munich, Munich, Germany; University of Ljubljana, SLOVENIA

## Abstract

**Introduction:**

COVID-19 is a multi-systemic disease which can target the lungs and the cardiovascular system and can also affect parts of the brain for prolonged periods of time. Even healthy athletes without comorbidities can be psychologically affected long-term by COVID-19.

**Objective:**

This study aimed to investigate athletes’ perceived mental stress and recovery levels in daily life, and their maximal aerobic power, at three different time points, post COVID-19.

**Methods:**

In total, 99 athletes (62.6% male), who had been infected by COVID-19, filled out the Recovery Stress Questionnaire for Athletes (REST-Q-Sport) and completed cardiopulmonary exercise testing (endpoint maximal aerobic power output (P_max_)) at the initial screening (t_1_: 4 months after infection). Follow-up assessments occurred three (t_2_, n = 37) and seven months after t_1_ (t_3_, n = 19).

**Results:**

Subgroup means from the Recovery category were significantly below the reference value of four at all three time points, except “General Recovery” (3.76 (± 0.96), p = 0.275, d = 0.968) at t_3_.”Overtiredness” (2.34 (± 1.27), p = 0.020, r = 0.224) was significantly above the reference value of two at t_1_, while all other Stress subgroups were not significantly different from the reference value or were significantly below the maximum threshold of two at t_1_, t_2_ and t_3_. Spearman’s ρ revealed a negative association between P_max_ and the subcategories of stress (ρ = -0.54 to ρ = -0.11, p < 0.050), and positive correlations between P_max_ and “Somatic Recovery” (ρ = 0.43, p < 0.001) and “General Recovery” (ρ = 0.23, p = 0.040) at t_1_. P_max_ (t_1_: 3.83 (± 0.99), t_2_: 3.78 (± 1.14), β = 0.06, p < 0.003) increased significantly from t_1_ to t_2_. In addition, REST-Q-Sport indicated a decrease in "Sleep" (t_2_ = 2.35 (± 0.62), t_3_ = 2.28(± 0.61), β = -0.18, p < 0.023) at t_3_, when compared to t_2_.

**Conclusion:**

The perceived recovery seems to be negatively affected in post COVID-19 athletes. Physical performance post COVID-19 correlates with both “Emotional and Somatic Stress” and “Somatic and General Recovery”, indicating potential mental and physical benefits of exercise. While it is evident that COVID-19, like other viral infections, may have an influence on physical performance, monitoring stress and recovery perceptions of athletes is critical to facilitate their return-to-sports, while minimizing long-term COVID-19 induced negative effects like the athletic objective and subjective perceived recovery and stress levels.

## 1. Introduction

The Coronavirus disease 2019 (COVID-19) has spread globally and resulted in a global health emergency and pandemic. To date, the long-term health repercussions of this pandemic (e.g., Post-COVID syndrome (persistent symptoms > 12 weeks from the onset of acute symptoms), dealing with and healing symptoms like fatigue, concentration problems) are not yet completely understood [1]. It is currently well established that, in addition to risk factors like age and the presence of chronic diseases, other factors, such as too low or too high body mass index (BMI), can put individuals at a higher risk for developing long-term post COVID-19 symptoms [2]. COVID-19 can lead to reduced mental and physiological performance, even among athletes who were previously in good physical condition [3–5]. Consecutively, decreased athletic performance of affected athletes can persist over a long period of time [6]. Changes in perception of stress and recovery influence performance [7–10]. Therefore, the mental health is especially important for athletes not only for daily life quality like in the general population, but also for training and competition performance.

COVID-19 is a multi-systemic disease [11] which can influence mental health by affecting different parts of the brain [12,13]. Neurological problems resulting from COVID-19 can persist over a long period of time [5]. Increased inflammatory processes and decreased anti-oxidative defense during the acute phase of COVID-19 have been observed, and may have a direct influence on post-viral somatic and mental symptoms of developing Long COVID [14]. Athletes generally benefit from the positive effects of exercise, for example a reduction of inflammatory markers and prevention of depression [15,16]. In contrast, however, large monotonous training volumes may increase stress [17]. The altered perception of stress can lead to the triggering of inflammatory processes [18]. The pandemic also resulted in a decrease in social activities, which may have led to a reduction of mental health among some individuals [19]. Persistent post COVID-19 symptoms (e.g. fatigue, concentration problems or shortness of breath) and chronic inflammation can lead to similar consequences as the overtraining syndrome, for example depressive symptoms or the deterioration of mental stability [4,15,20,21].

Additional stressors experienced by athletes include competitions, social and environmental factors, and imbalances between training and regeneration, which can all lead to stress and an altered stress resilience [22]. This can result in unexpected performance loss [21,23], fatigue symptoms, hormonal disturbances, and increased pro-inflammation and immune system activation [15,18]. Due to the close relationship between mental and physical factors and athletic performance, Kellmann et al. [24] developed a questionnaire that explicitly addresses stress and recovery factors among athletes. Persistent stress and sustained insufficient recovery can result in reduced performance [25]. Consequently, these factors should be considered, when monitoring recovery from COVID-19 among recently infected athletes. However, long-term monitoring of mental well-being is rare for post-COVID-19 athletes, and the connections between mental health and athletic performance of post-COVID-19 athletes have not yet been sufficiently observed.

The three aims of this study focused on the investigation of the altered stress and recovery perception of athletes in relation to their maximal aerobic power after COVID-19. The first aim was to investigate the relationship between physical performance and the subgroups of stress and recovery in daily life of athletes post COVID-19. Secondly, we examined which covariates influence the variables of REST-Q-Sport, and the correlation between maximal aerobic power and the REST-Q-Sport variables. Subsequently, in a long-term observation up to seven months, the development of the subgroups of stress and recovery, and athletic performance was analyzed.

## 2. Methods

### 2.1 Study population

The study was conducted at the Division of Sports and Rehabilitation Medicine, Centre of Internal Medicine of University Hospital Ulm, Germany. All data were collected as a part of the CoSmo-S study (COVID-19 in German Competitive Sports) at the University Hospital Ulm [26].

The data was collected between December 2020 and May 2022. Inclusion criteria were the following: 1) age ≥ 18 years, 2) regular physical training with a minimum of three training sessions per week (minimum of 20 METs/week), 3) post COVID-19 state (verified by result of positive smear-PCR and/or positive antigen testing). A detailed description of all inclusion and exclusion criteria, as well as the study design, can be found in the study protocol by Nieß et al. [26].

All patients who filled out the Recovery Stress Questionnaire for Athletes (REST-Q-Sport) by Kellmann et al. [24] and completed cardiopulmonary exercise testing at their first appointment were included in our pilot evaluation. Assessments of follow-up measurements are still ongoing.

In total, 99 participants (62.6% male) were included for initial assessment. A detailed description of the study population, including, sport type, pre-disease training volume, symptoms during acute phase as well as persistent symptoms at each examination are shown in Table 1.

**Table 1:** Type of sports, pre-disease training volume per week, duration and kind of symptoms during acute phase, persistent symptoms at each examination, as well as possible impairments for performance due to other diseases or comorbidities. Multiple answers were possible for the types of sports and for the persistent symptoms.

**Sport**	**N(99)**					
*Type of sport*:	**Endurance**	**Resistance**	**Team / Combat**	**Technique**	**Missing**	
*Number*	55	20	35	11	1	
Volume per week*	**≤ 5h**	**> 5–10h**	**> 10–15h**	**> 15h**	**Missing**	
*Number*	44	29	12	11	3	
**Symptoms in acute phase**	**N(99)**					
	**1–3 days**	**4–6 days**	**1–2 weeks**	**Longer than 2 weeks**	**No**	**Missing**
*Fever (>38°C)*	28	6	4	1	41	19
*Cough*	10	10	12	11	37	19
*Ageusia / Anosmia*	11	5	6	16	41	20
*Rhinitis*	17	15	10	4	34	19
*Throat pain*	23	9	7	2	39	19
*Dyspnea under load*	5	5	6	14	50	19
*Dyspnea at rest*	5	6	3	2	64	19
*Diarrhea*	10	2	2	1	65	19
*Headache*	15	10	8	10	35	21
Persistent symptoms**	**t**_**1**_ **N(99)**	**t**_**2**_ **N(37)**	**t**_**3**_ **N(19)**			
*Fatigue and performance decrease*	51	14	9			
*Sleeping disorders*	27	10	4			
*Neurocognitive disorders*	31	13	10			
*Respiratory disorders*	32	6	5			
*Autonomic disorders*	21	6	5			
*Muscle pain*	12	4	3			
*Psychological-related items*	4	4	1			
*Immunological disorders*	4	3	3			
*No symptoms*	36	14	8			
**Possible impairments caused by**:	**Obesity**	**Pulmonary**	**Cardio-vascular**	**Systemic**	**Others**	
	8	13	13	4	5	

* Training volume before disease, ** Symptoms categories.

**Fig 1:**
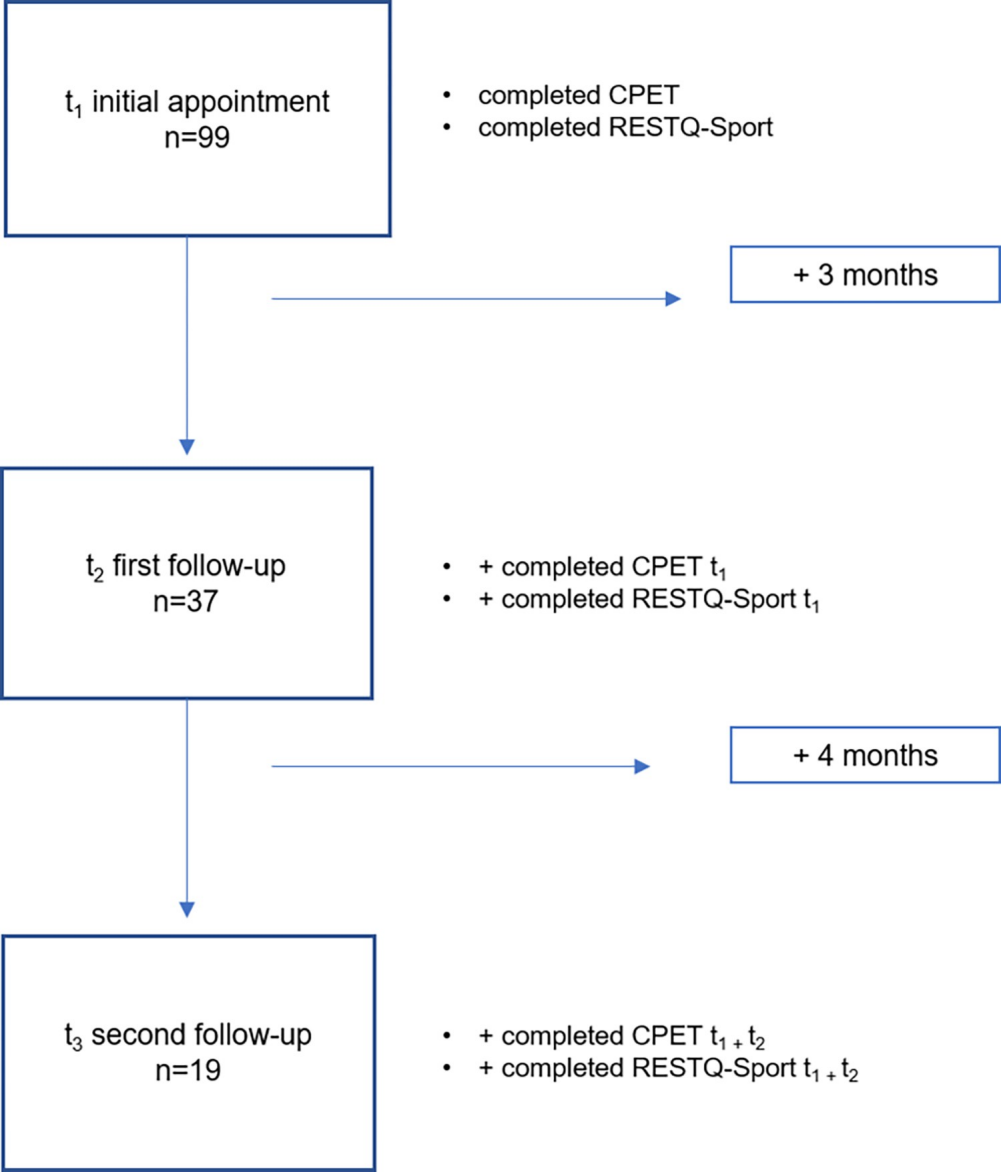
Flow-Chart of study population of each time point. CPET (cardiopulmonary exercise testing), REST-Q-Sport (Recovery and Stress Questionnaire for athletes).

The mean time between acute infection and initial screening (t_1_) was 3.93 (± 4.41) months, with a median value of 2.5 months. The second examination (t_2_) took place three months later (3.08 ± 0.30). The third examination t_3_ was seven months after t_1_ (6.92 ± 0.25). In total, 37 athletes were included in the analysis considering t_1_+t_2_, and n = 19 when including t_3_. Only athletes with complete data sets were included in the follow-up evaluations (Fig 1).

### 2.2 Examination

To assess recovery and stress level in daily life at t_1_, t_2_, and t_3_, participants were asked to fill out the German version of the REST-Q-Sport after Kellmann et al. [24]. The REST-Q-Sport consists of 76 items which assess the following four categories: Stress, Recovery, Injury and Performance. Participants were asked to base their responses to the questions on the last three days and answers were scored with a 7-point-Likert-scale, 0 = never to 6 = always. The individual items could be assigned to 19 factors (subgroups). To determine the subgroups values, the mean value of the corresponding items was calculated. In this context, four questions were needed per subgroup to determine a mean value. A mean value above four for the Recovery and Performance subgroups and below two for the Stress and Injury subgroups were considered as trivial [24]. The categories Stress and Recovery were considered in detail in the evaluation section of this study, whereas the categories Injury and Performance were detailed in S1 and S2 Tables in S1 File. Within the Recovery category, the subgroups “Success”, “Social Recovery”, “Somatic Recovery”, “General Recovery”, and “Sleep” (refers to sleep quality) were assessed, respectively. For Stress, “General Stress”, “Emotional Stress”, “Social Stress”, “Performance Pressure”, “Overtiredness”, “Lack of Energy”, and “Somatic Stress” were analyzed.

Additionally, all participants conducted cardiopulmonary exercise testing (CPET). CPET was used to determine the relative maximal aerobic power (P_max_), displayed as W/kg Body Mass (BM), which was measured on a bicycle ergometer (Lode Corival and Lode Excalibur, Lode B.V., Groningen, Netherlands).

Each participant completed a standardized ramp exercise protocol to exhaustion. After a warm-up of two minutes of unloaded pedaling, the power output was increased between 15 W/min and 40 W/min, depending on fitness and biological sex. Based on current symptoms, previous testing, or training status, the respective protocol was chosen to achieve total exhaustion within eight to twelve minutes.

Anthropometric measurements included height, body weight, P_max_ and lean body mass (Table 2). For measuring body mass and body fat, a bio-impedance scale (InBody 770, InBody Europe B.V., Eschborn, Germany) was used. Lean Body Mass (LBM) was used to diminish differences between genders.

**Table 2:** Anthropometric characteristics of the study population at t_1_.

	N	Mean (± SD)
**Age (years)**	99	33.0 (11.9)
**Body mass (kg)**	97	75.2 (14.3)
**Height (cm)**	97	176.9 (9.0)
**BMI (kg/m** ^**2**^ **)**	96	23.9 (3.5)
**LBM (kg)**	88	56.6 (15.2)
**P**_**max**_ **(W/kg BM)**	99	3.8 (1.0)

Differences in data collection numbers are due to omissions in primary data collection or invalid samples. BMI (body mass index), LBM (lean body mass), P_max_ (maximal aerobic power / kg BM).

### 2.3 Statistical analysis

Data analysis was performed using the GraphPad Prism (©GraphPad Prism 9.3.1.4), SPSS (©IBM SPSS Statistics, version 28.0.0.0 (190)), and R version 4.1.2 (R Core Team, 2021) software.

Descriptive values were displayed as mean values and standard deviations (± SD). Differences between the means and the corresponding reference values were determined using T-test or Wilcoxon test. Effect size was calculated with Cohen’s d or r, respectively.

Correlation analysis between the subgroups of Recovery and Stress and P_max_ was performed using Spearman’s ρ. The influence of the covariates (age, body mass, BMI, LBM and time since infection), which showed a significant correlation to P_max_ and Stress subgroups, was considered in the partial correlation.

To calculate whether the parameters changed from the initial screening t_1_ to the first follow-up t_2_ and from t_1_ to t_3_, a multilevel mixed models were used. The calculations were corrected for multiple testing using fdr-correction.

Robust linear regression was calculated to capture the influence of time since acute infection on the Recovery and Stress subgroups.

The significance level for all tests was set at p<0.050.

### 2.4 Ethics

All athletes were provided with information about the content of the study and the use of the data, and provided written consent. The study was conducted in compliance with the Declaration of Helsinki. The study was approved by the ethics committee of Ulm University (EK-408/20).

## 3. Results

### 3.1 REST-Q-Sport and P_max_ compared to reference values

Tables 3 and 4 show the mean and median values of the subcategories of Recovery and Stress and whether they differed significantly from the reference values, also including the effect size. All Recovery subgroups were significantly lower compared to the reference value four, except “General Recovery” at t_3_. Thus, a large part of the questionnaire data given by the athletes were not in the desirable range. The effect size varies from medium to strong |-0.356–1.231|.

**Table 3:** Mean and median values as well as the effect size and p-value of subcategories of Recovery for first appointment t_1_ (n = 99), second appointment t_2_ with completed data sets of t_1_ and t_2_ (n = 37), and third appointment t_3_ with completed data sets of t_1_, t_2_, and t_3_ (n = 19). Significance level p < 0.05.

t_1_	N	Mean (± SD)	Median	Effect size (p-value)
**Success**	99	2.3 (± 1.1)	2.3	**1.052 (p<0.001)**
**Social Recovery**	99	3.0 (± 1.2)	3.0	**1.231 (p<0.001)**
**Somatic Recovery**	99	2.8 (± 1.2)	2.8	**1.201 (p<0.001)**
**General Recovery**	99	3.5 (± 1.2)	3.5	**1.198 (p<0.001)**
**Sleep**	99	2.5 (± 0.7)	2.5	**-0.857 (p<0.001)**
**t**_**2**_				
**Success**	37	2.5 (± 1.1)	2.5	**1.094 (p<0.001)**
**Social Recovery**	37	3.0 (± 1.1)	**3.3**	**-0.730 (p<0.001)**
**Somatic Recovery**	37	2.2 (± 1.4)	**3.8**	**-0.492 (p = 0.003)**
**General Recovery**	37	3.6 (± 1.2)	**3.8**	**-0.356 (p = 0.030)**
**Sleep**	37	2.4 (± 0.6)	**2.5**	**-0.874 (p<0.001)**
**t**_**3**_				
**Success**	19	2.6 (± 0.8)	**2.8**	**0.782 (p<0.001)**
**Social Recovery**	19	3.0 (± 1.2)	**3.3**	**-0.661 (p = 0.004)**
**Somatic Recovery**	19	3.3 (± 1.1)	**3.5**	**1.104 (p = 0.013)**
**General Recovery**	19	3.8 (± 1.0)	3.8	0.968 (p = 0.275)
**Sleep**	19	2.3 (± 0.6)	2.3	**0.569 (p<0.001)**

**Table 4:** Mean and median values as well as the effect size and p-value. of subcategories of Stress for first appointment t_1_ (n = 99), second appointment t_2_ with completed data sets of t_1_ and t_2_ (n = 37), and third appointment t_3_ with completed data sets of t_1_, t_2_, and t_3_ (n = 19). Significance level p < 0.05.

t_1_	N	Mean (± SD)	Median	Effect size (p-value)
**General Stress**	99	1.5 (± 1.2)	1.5	**-0.341 (p<0.001)**
**Emotional Stress**	99	1.7 (± 0.9)	**1.5**	**-0.358 (p<0.001)**
**Social Stress**	99	1.5 (± 0.9)	1.5	**-0.476 (p<0.001)**
**Performance Pressure**	99	1.8 (± 1.0)	1.8	**-0.254 (p = 0.012)**
**Overtiredness**	99	2.3 (± 1.3)	2.3	**0.224 (p = 0.020)**
**Lack of Energy**	99	2.3 (± 1.2)	2.3	0.169 (p = 0.093)
**Somatic Stress**	99	2.0 (± 1.3)	2.0	-0.041 (p = 0.686)
**t**_**2**_				
**General Stress**	37	1.6 (± 1.3)	1.0	-0.283 (p = 0.085)
**Emotional Stress**	37	1.6 (± 0.9)	1.5	**-0.415 (p = 0.016)**
**Social Stress**	37	1.7 (± 0.9)	1.5	-0.277 (0.092)
**Performance Pressure**	37	1.9 (± 1.2)	1.8	-0.107 (p = 0.518)
**Overtiredness**	37	2.2 (± 1.3)	2.0	1.315 (p = 0.477)
**Lack of Energy**	37	2.1 (± 1.3)	2.0	1.305 (p = 0.685)
**Somatic Stress**	37	1.9 (± 1.4)	1.8	1.399 (p = 0.705)
**t**_**3**_				
**General Stress**	19	1.6 (± 1.3)	1.5	1.319 (p = 0.157)
**Emotional Stress**	19	1.6 (± 1.1)	1.5	1.377 (p = 0.126)
**Social Stress**	19	1.6 (± 0.8)	**1.8**	0.813 (p = 0.064)
**Performance Pressure**	19	2.0 (± 1.2)	**2.3**	1.176 (p = 0.885)
**Overtiredness**	19	2.2 (± 1.7)	**2.0**	0.033 (p = 0.887)
**Lack of Energy**	19	2.2 (± 1.5)	**1.3**	0.065 (0.776)
**Somatic Stress**	19	1.8 (± 1.5)	**1.3**	1.542 (p = 0.584)

The values of the Stress subgroups “General Stress” (r = -0.341 (p < 0.001)), “Emotional Stress” (r = -0.358 (p < 0.001)), “Social Stress” (r = -0.476 (p < 0.001)) and “Performance Pressure” (r = -0.254 (p = 0.012)) were significant lower compared to the reference value of two at t_1_, whereas “Overtiredness” (r = 0.224 (p = 0.020)) was increased at t_1_. At t_2_ “Emotional Stress” (d = -0.415 (p = 0.016)) was significantly lower than the reference value. Thus, this value is within the desired range. At t_3_, there were no significant differences between the subgroups of Stress and the reference value.

The values of P_max_ were between 3.5 and 3.9 W/kg BM (t_1_: 3.83 (± 0.99), t_2_: 3.78 (± 1.14), t_3_: 3.50 (± 1.10)). Significant differences between genders were not observed. The mean and median values of the subgroups in the Injury category were all within the reference range, while the mean and median values of the subgroups in the Performance category were below the reference values. Additional results of the Injury and Performance categories are provided in S1 and S2 Tables in S1 File.

### 3.2 Association between REST-Q-Sport and P_max_

**Fig 2:**
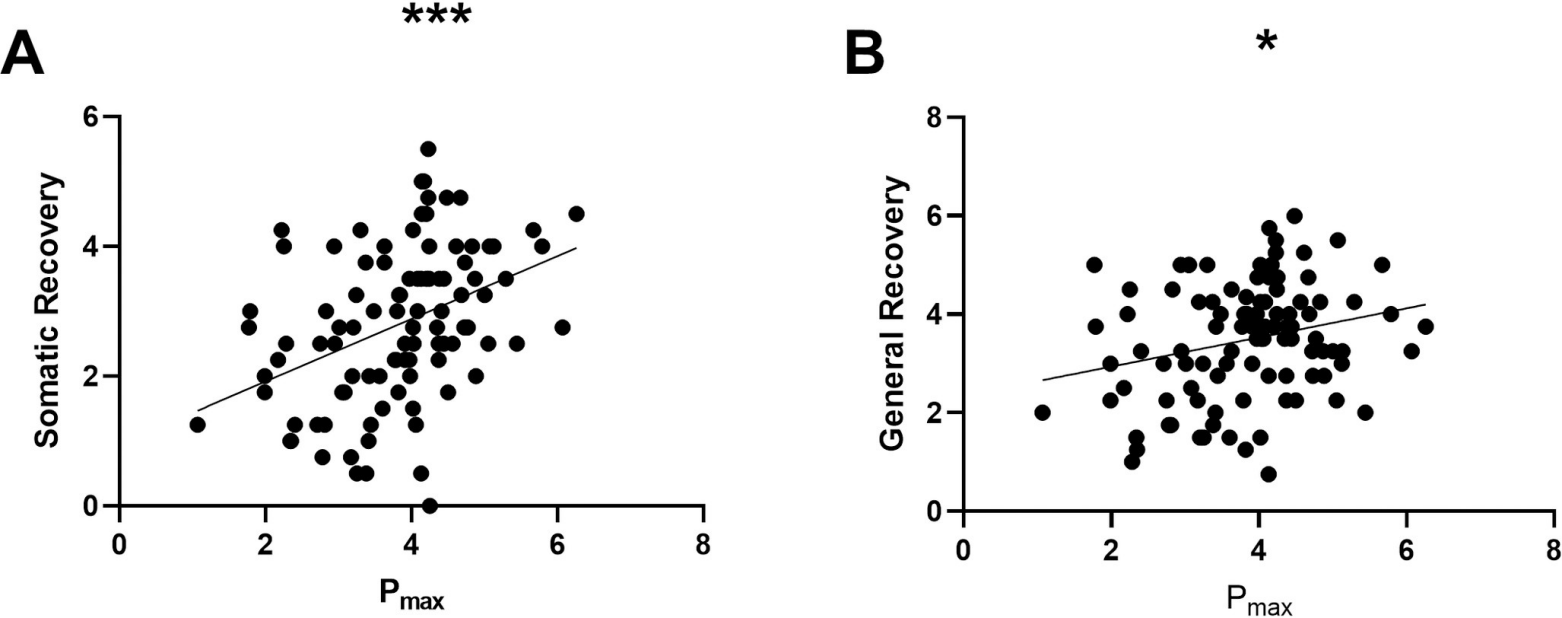
Positive correlations between P_max_ (maximal aerobic power W/kg BM) and Somatic Recovery (A) and General Recovery (B). *p<0.05, **p<0.01, ***p<0.001 p* = correlation with P_max_ at t_1_, controlled for multiple testing.

**Fig 3:**
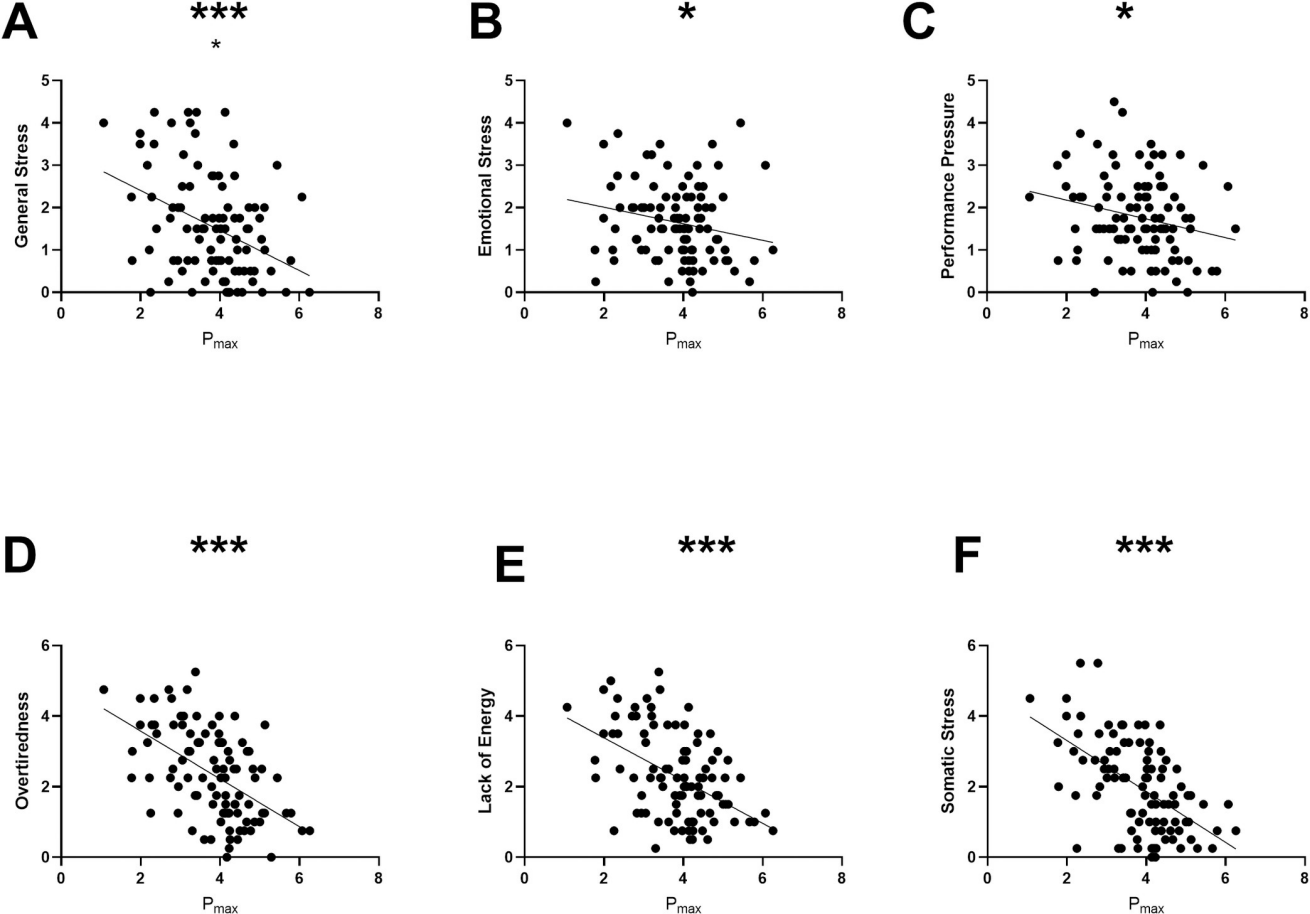
Negative correlations between P_max_ (maximal aerobic power W/kg BM) and General Stress (A), Emotional Stress (B), Performance Pressure (C), Overtiredness (D), Lack of Energy (E) and Somatic Stress (F). *p<0.05, **p<0.01, ***p<0.001, p* = correlation with P_max_ at t_1,_ controlled for multiple testing.

Spearman’s rank correlations between P_max_ and”Somatic Recovery” and “General Recovery”, and P_max_ “General Stress”, “Emotional Stress”, “Performance Pressure”, “Overtiredness”, “Lack of Energy” and “Somatic Stress”, were displayed in Figs 2 and 3. Individuals with higher P_max_ showed significantly higher “Somatic Recovery” (ρ = 0.43, p < 0.001) and “General Recovery” (ρ = 0.23, p < 0.036). No significant association between “Success”, “Social Recovery”, and “Sleep” and P_max_. was found. Negative correlations were observed between P_max_ and “Emotional Stress” (ρ = -0.21, p = 0.053), “Performance Pressure” (ρ = -0.23, p<0.035), “Overtiredness” (ρ = -0.52, p < 0.001), “Lack of Energy” (ρ = -0.45, p<0.001) and “Somatic Stress” (ρ = -0.54, p<0.001). After correcting for multiple testing, all results remained unchanged. A detailed analysis of the correlation between each subgroup can be found in S3 Table in S1 File.

#### 3.2.1 Adjusting for covariates

Significant correlations between the covariates age, BMI, LBM, sex, body mass and time since infection at t_1_ were shown in Table 5. The correlations between the parameters were not significantly affected by the covariates (S4–S15 Tables in S1 File). However, a significant influence of the covariates on the individual parameters occurred (Table 5).

**Table 5:** Significant influences of covariates on targeted subgroups at t_1_.

Covariate	Assessed variable	Rho-value	p-value
**LBM**			
	P_max_	0.55	0.001
	General Stress	-0.21	0.046
	Success	0.22	0.035
**BMI**			
	P_max_	-0.43	0.001
**Age**			
	Success	0.22	0.039
	Social Recovery	-0.31	0.002
	Sleep	0.26	0.014
**Time since infection**			
	Sleep	-0.23	0.025

Significance level: p<0.050. Rho shows the size of the positive / or negative effect on the respective subgroup.

### 3.3 Change of REST-Q-Sport subgroups three and seven months after initial appointment

#### 3.3.1 Change of subgroups between t_1_ and t_2_

For the 37 athletes with complete data sets at t_1_ and t_2_, a significant change of P_max_ was observed with an improvement in P_max_ over the three-month period (β = 0.06 (0.02–0.10), p < 0.003). The parameters of the REST-Q-Sport did not show any significant changes over the first three months.

#### 3.3.2 Change of subgroups between t_1_, t_2_ and t_3_

A complete data set over a period of seven months was collected from 19 athletes. No significant change in P_max_ could be shown. Moreover, there was no significant change in the subcategories of Stress. Participants did not show a significant change in the perception of recovery besides “Sleep”. The perception of “Sleep” decreased between the first and the third appointment (β = -0.18, [-0.33–0.02], p < 0.023).

## 4. Discussion

In the present study, higher levels of decreased perceived recovery were observed among individuals who had recently COVID-19. Furthermore, correlations were found between the subgroups of the REST-Q-Sport and the maximum aerobic performance. Higher values in “General Stress”, “Emotional Stress”, “Performance Pressure”, “Overtiredness”, “Lack of Energy” and “Somatic Stress” were associated with lower P_max_. Higher values of P_max_ correlated positively with the Recovery subgroups “Somatic Recovery” and “General Recovery”. When examining changes over three and seven months, an improvement of P_max_ between t_1_ and t_2_ and a decrease in “Sleep” after a period of seven months could be observed.

### 4.1 Differences between Stress / Recovery subgroups in regard to the reference values

On the one hand, all mean values of the Recovery subgroups were below the desirable range. Only "General Recovery" was not significantly below the threshold at t_3_. On the other hand, only "Overtiredness" from the Stress category significantly exceeded the threshold of two. These results suggest that perceived recovery is frequently disturbed. Small disturbances occur in daily life [27]. Healthy individuals can cope with these disturbances quickly. Coping ability are delayed in people with symptoms. In the long term, the reduced coping ability may lead to slower recovery and thus to a reduced resilience [27]. COVID-19 could be such a trigger: affected individuals have slower processing time. However, this assumption requires further research.

### 4.2 Correlation between Stress / Recovery and P_max_ after COVID-19

#### 4.2.1 Correlation between Recovery and P_max_

Kalda et al. [28] observed that “General Recovery” is positively associated with enhanced performance during competitions. This is consistent with our findings, as we found a positive correlation between higher P_max_ values and improved perception of “General Recovery” in post COVID-19 athletes. We also found that an increased perception of “Somatic Recovery” correlated with enhanced physical performance.

#### 4.2.2 Correlation between Stress and P_max_

In the present study, elevated “General Stress”, “Emotional Stress” and “Performance Pressure” negatively impacted performance after four months of infection. To date, there are no official recommendations for athletes suffering from long-term symptoms post COVID-19 [29]. Wilson et al. [30] recommended a similar management of the return to sport after COVID-19 as the guidelines for myocarditis. Additional studies found that training volume was reduced during lockdown durations, regardless of the level at which the athlete was exercising [31,32]. Furthermore, most athletes trained alone and the training intensity in team sports decreased more compared to other sports [31,32]. These results can also be applied to our study and might explain why "Fitness / Being in shape" and "Social Recovery" were rated low.

A further study group compared the mental health status of athletes and non-athletes and did not find any differences in stress and anxiety levels during the pandemic [33]. However, they found that athletes who were currently injured had a higher stress and anxiety score than non-injured athletes. This is in line with our findings, that “General Stress”, “Emotional Stress”, and “Performance Pressure” were elevated among post COVID-19 athletes. However, an increase in perceived stress can also be triggered by competitions, which is a factor which specifically applies to athletes [34].

Steinacker et al. [35] have already observed that high stress levels during high training volume phases can affect central (e.g. hypothalamic sympathetic dysregulation) and peripheral mechanisms (e.g. hematological and metabolic deficits). Furthermore, an increase in general emotional and physiological stress was seen in elite athletes during competition phases, presumably leading to an alerted immune response and inflammatory processes that might be interconnected with mental and physical performance [18]. Therefore, it can be assumed that higher stress levels in post COVID-19 athletes can lead to an altered immune system, which can trigger inflammatory processes. However, it is also possible that altered inflammatory processes can lead to higher stress levels [36]. Hence, further research is needed to investigate this possible interdependence and the disease origin in the respective patient.

Another health concern of COVID-19 is sleep disturbance, which may have its origin in mental health problems, which leads to poor sleep quality and thus to overtiredness [8,37]. Although we did not find a correlation between “Sleep” and P_max_, we found a negative correlation between “Overtiredness” and P_max_. It was already observed that, among athletes where the training intensity decreased during the lockdowns, the sleep quality also simultaneously decreased [38]. These results might indicate that people who are no longer active, due to persistent symptoms, tend to have poorer sleep quality. Furthermore, persistent symptoms can lead to other life changes, such as job loss or changes in personal life, which can also affect sleep quality. Since sleep quality obviously has a major impact on daily performance but is also influenced by many other factors, further research should be conducted on the long-term effects of COVID-19 on sleep quality.

Brink et al. [39] showed in their study that emotional stress can be interpreted as an early warning parameter for declining performance. The subgroup “Emotional Stress” also had a negative influence on P_max_ in our study.

In general, Long-COVID or Post-COVID challenges mental well-being, and the return to sport can add challenging circumstances, especially when long-term symptoms occur [40]. With this in mind, mental health must be considered in individualized training plans. Without individualized training counseling there is the potential risk that a short-term decline in performance after an acute infection can lead to a long-term decline in performance [41].

#### 4.2.3 Covariates for performance

As presented in this study, body composition influences maximal aerobic power. Our athletes with adequate lean body mass showed better performance capability, when compared to athletes below or above reference values. In addition, we showed a negative correlation between LBM and General Stress. These results are in line with previous literature, where it is well established that inadequate body composition can be associated with mental health problems like depression, especially in women [42]. However, other factors can also promote depression.

It is important to note that the pandemic interrupted social interactions, togetherness, and familiar (social) routines. Additionally, for our athletic sample in particular, these restrictions can be a striking experience, as sport always has a strong social component, which was largely eliminated in the pandemic.

### 4.3 Change of REST-Q-Sport subgroups three and seven months after initial appointment

It is well established that COVID-19 can lead to long-term sequela [43–45]. These limiting and long-term symptoms can decrease quality of life [46] and are therefore key parameters when monitoring post COVID-19 patients.

After the first follow-up, our results showed a significant improvement of P_max_ and no change in the perceptions of Stress and Recovery.

Over the seven-month period, however, no significant change of P_max_ could be determined. Generally, after the acute phase of a disease, performance increases to the level before infection, when restarting with sport. This may explain the increase in performance between the first and the second appointment. Over time, the influence of the original infection becomes smaller, and the influence of regular training becomes greater. The results of the study suggest that in this population there may no progressive training, or that a leveling off of performance had already been achieved and the effects of the infection were minor.

Betschart et al. [45] showed deteriorated performance among 50% of their study participants, while the other half improved (determined by walking distances). These findings highlight the individuality of the recovery process, which we also observed with regards to persistent symptoms and performance.

After the second follow-up, a decrease in perceived “Sleep” was observed. Sleep is a crucial factor when monitoring general recovery and should be assessed with associated parameters (duration, quality, individual circadian rhythm etc.), especially within the field of sports [47]. Furthermore, not only the disease itself, but also the lockdowns caused by the pandemic had effects on sleep quality [48]. Therefore, altered sleep pattern can be caused by a variety of reasons.

Donzella et al. [49] showed that individuals who had COVID-19 reported lower sleep quality than individuals who did not have COVID-19, although sleep time was higher. Hausswirth et al. [50] demonstrated in their study that functionally overreached athletes had a decline in sleep efficacy and 50% of them had a performance loss after six weeks. The observed decreased sleep efficacy and decrease in performance [50] are similar to long-term symptoms which can occur post COVID-19 [5,51]. Therefore, we assume that overtraining and COVID-19 may altered the immune system and physical functions in a similar way.

## 5. Strengths and limitations

To our knowledge, there are no comparable studies that have investigated the relationship between physical performance and mental parameters in post COVID-19 athletes. The short time span in the questionnaire that we focused on is also a strength of our study because it highlights even small changes in perceptions of stress and recovery, rather than focusing on states that are quite stable and change only slowly over time. Nevertheless, the results of the study cannot establish a direct connection between the illness and the altered perceived stress and recovery, as no measurement data were available before the disease. Furthermore, the sport type and weekly training volume differed within the study population. In general, however, these individuals were more physical active than the average German population compared to our study population in the same age group (33.0 yrs). Therefore, we could assume that this study population was fitter and had fewer comorbidities.

Our results indicate that especially the perceived recovery is affected. Due to an increasing dropout rate over time, the power of the results decrease. In addition, it should be noted that the willingness to participate in the study was probably greater among individuals with existing symptoms than symptom-free athletes. However, the percentage of athletes who were symptom-free at examination increased slightly at each examination. This fact may explain why there were almost no significant changes over time. With a larger study population, group divisions (e.g., into different persistent symptoms) and comparisons would be possible.

Furthermore, we did not take weekly training hours, sleep time, or inflammatory biomarkers into account, which could be confounding components when detecting perceived stress and recovery. Furthermore, we did not investigate whether athletes had COVID-19 again, and we did not monitor their exercise behavior over time.

### 6. Conclusion

In our post COVID-19 athletic study population, a relationship between maximal aerobic power and perceived stress and recovery was observed. Furthermore, insufficient recovery and slightly increased stress parameters were observed at all examination points. Together with the detrimental effects of “Sleep” for a long-term decrease in performance, the importance of monitoring not only physical but also psychological parameters post COVID-19 athletes is highlighted and should be further integrated in continuous routine documentation by athletes and physicians. The combination of 1) performance testing (CPET), 2) mental state assessment (REST-Q-Sport) and 3) molecular biomarkers (inflammatory/immunological) may support in the assessment of the respective athlete’s health status. In addition, this combination may assist in the development of individualized training plans which also consider mental well-being, to help the athletes to return to sport, improve their performance, and attenuate long-term consequences.

## Supporting information

S1 File Subcategories of Injury and Performance, correlation matrix and influences of covariates. (DOCX)

## Abbreviations

BM: Body Mass
BMI: Body mass index (kg/m^2^
CoSmo-S: COVID 19 in German Competitive Sports
CPET: Cardiopulmonary Exercise Testing
LBM: lean body mass
P_max_: Maximal aerobic Power / kg body mass
REST-Q-Sport: Recovery Stress Questionnaire for Athletes
t_1_: initial examination
t_2_: first follow-up 3 months post initial examination
t_3_: second follow-up 7 months post initial examination
