# Supplementary material for: Relationship between physical performance and perception of stress and recovery in daily life post COVID-19—An explorative study
Source: PLoS One. 2023 May 15;18(5):e0285845. doi: 10.1371/journal.pone.0285845 (PMC10184944; doi:10.1371/journal.pone.0285845)
Supplement: S1 File — (DOCX) [file pone.0285845.s001.docx]

**Supplemental**

Table 1. Mean and median values of subcategories of Injury for first appointment t_1_ (n=99), second appointment t_2_ with completed data sets of t_1_ and t_2_ (n=37), and third appointment t_3_ with completed data sets of t_1_, t_2_, and t_3_ (n=19). Median bolded when difference to mean >0.1.

| **Injury** | **t_1_**  **Mean (±** **SD)** | **t_1_**  **Median** | **t_2_**  **Mean (± SD)** | **t_2_**  **Median** | **t_3_**  **Mean (± SD)** | **t_3_**  **Median** |
| --- | --- | --- | --- | --- | --- | --- |
| **Disturbed Break** | 1.43 (±0.99) | **1.25** | 1.67 (± 1.00) | **1.50** | 1.64 (±0.99) | **1.50** |
| **Burnout/ emotional Fatigue** | 1.38 (±1.28) | **1.00** | 1.30 (± 1.28) | **1.00** | 1.30 (±1.28) | **1.13** |
| **Fitness/ Injury susceptibility** | 1.58 (±1.20) | **1.25** | 1.52 (± 1.11) | **1.38** | 1.40 (±1.20) | 1.38 |

Table 2. Mean and median values of subcategories of Performance for first appointment t_1_ (n=99), second appointment t_2_ with completed data sets of t_1_ and t_2_ (n=37), and third appointment t_3_ with completed data sets of t_1_, t_2_, and t_3_ (n=19). Median bolded when difference to mean >0.1.

| **Performance** | **t_1_**  **Mean (±** **SD)** | **t_1_**  **Median** | **t_2_**  **Mean (± SD)** | **t_2_**  **Median** | **t_3_**  **Mean (± SD)** | **t_3_**  **Median** |
| --- | --- | --- | --- | --- | --- | --- |
| **Fitness/being in shape** | 2.74 (±1.25) | **2.50** | 3.10 (± 1.38) | **3.50** | 3.16 (±1.25) | **3.38** |
| **Burnout/Personal Realization** | 2.41 (±1.41) | 2.50 | 2.44 (± 1.33) | 2.50 | 2.66 (±1.41) | 2.75 |
| **Self-efficacy beliefs** | 2.42 (±1.36) | **2.25** | 2.73 (± 1.40) | 2.75 | 2.98 (± 1.36) | 2.88 |
| **Self-regulation** | 2.68 (±1.57) | **2.50** | 2.88 (± 1.53) | **3.00** | 3.19 (±1.57) | **3.38** |

Table 3. Correlation Matrix of target parameters of Stress and Recovery and P_max_ (Maximal Power W/kg BM).at t_1_.

|  | **General Stress** | **Emotio-nal Stress** | **Social Stress** | **Perfor-mance Pressure/Conflicts** | **Over-tired-ness** | **Lack of Energy** | **Somatic Stress** | **Suc-cess** | **Social Recovery** | **Somatic Recovery** | **General Recovery** | **Sleep** | **P_max_** |
| --- | --- | --- | --- | --- | --- | --- | --- | --- | --- | --- | --- | --- | --- |
| **Emotional Stress** | **.548^**^** |  |  |  |  |  |  |  |  |  |  |  |  |
| **Social Stress** | **.501^**^** | **.569^**^** |  |  |  |  |  |  |  |  |  |  |  |
| **Performance Pressure/Conflicts** | **.442^**^** | **.465^**^** | **.418^**^** |  |  |  |  |  |  |  |  |  |  |
| **Over-tiredness** | **.425^**^** | **.252^**^** | **.267^**^** | **.276^**^** |  |  |  |  |  |  |  |  |  |
| **Lack of Energy** | **.549^**^** | **.441^**^** | **.343^**^** | **.351^**^** | **.500^**^** |  |  |  |  |  |  |  |  |
| **Somatic Stress** | **.578^**^** | **.388^**^** | **.321^**^** | **.374^**^** | **.491^**^** | **.558^**^** |  |  |  |  |  |  |  |
| **Success** | **-.165^*^** | **-.209^**^** | **-.159^*^** | .074 | .002 | **-.170^*^** | -.131 |  |  |  |  |  |  |
| **Social Recovery** | **-.181^*^** | **-.184^*^** | -.122 | -.067 | -.107 | **-.244^**^** | **-.142^*^** | **.299^**^** |  |  |  |  |  |
| **Somatic Recovery** | **-.456^**^** | **-.440^**^** | **-.337^**^** | **-.303^**^** | **-.355^**^** | **-.478^**^** | **-.545^**^** | **.324^**^** | **.389^**^** |  |  |  |  |
| **General Recovery** | **-.503^**^** | **-.479^**^** | **-.364^**^** | **-.317^**^** | **-.286^**^** | **-.456^**^** | **-.364^**^** | **.332^**^** | **.466^**^** | **.539^**^** |  |  |  |
| **Sleep** | .029 | -.118 | -.083 | -.067 | .046 | .039 | .042 | .110 | **.194^**^** | **.158^*^** | **.227^**^** |  |  |
| **P_max_** | **-.254^**^** | **-.159^*^** | -.082 | **-.166^*^** | **-.374^**^** | **-.309^**^** | **-.381^**^** | .059 | .033 | **.303^**^** | **.151^*^** | -.069 |  |

Table 4. Robust linear Regression. Influence of time since infection and the covariates on P_max_ (Maximal Power W/kg BM) at t_1_.

|  | **P_max_** |  |  |  |  |
| --- | --- | --- | --- | --- | --- |
| *Predictors* | *Estimates* | *std. Beta* | *CI* | *p* | *df* |
| (Intercept) | 4.22 | 4.37 | 3.91 – 4.54 | **<0.001** | 95.00 |
| time since infect | -0.09 | -0.11 | -0.14 – -60.03 | **0.002** | 95.00 |
| R^2^ | 0.140 |  |  |  |  |
| (Intercept) | 3.55 | 3.11 | 2.68 – 4.43 | **<0.001** | 94.00 |
| time since infect | -0.08 | -0.05 | -0.14 – -0.02 | **0.006** | 94.00 |
| LBM | 0.01 | 0.02 | -0.00 – 0.03 | 0.122 | 94.00 |
| R^2^ | 0.184 |  |  |  |  |
| (Intercept) | 04.01 | 4.49 | 3.21 – 4.81 | **<0.001** | 94.00 |
| time since infect | -0.08 | -0.12 | -0.15 – -0.01 | **0.027** | 94.00 |
| age | -0.01 | -0.02 | -0.03 – 0.02 | 0.625 | 94.00 |
| R^2^ | 0.071 |  |  |  |  |
| (Intercept) | 6.93 | 6.25 | 5.49 – 8.36 | **<0.001** | 91.00 |
| time since infect | -0.07 | -0.10 | -0.12 – -0.03 | **0.003** | 91.00 |
| BMI | -0.12 | -0.08 | -0.18 – -0.06 | **<0.001** | 91.00 |
| R^2^ | 0.219 |  |  |  |  |
| (Intercept) | 3.66 | 4.63 | 3.04 – 4.29 | **<0.001** | 94.00 |
| time since infect | -0.07 | -0.13 | -0.15 – 0.00 | 0.060 | 94.00 |
| Sex | 0.79 | -0.34 | 0.16 – 1.43 | **0.015** | 94.00 |
| R^2^ | 0.311 |  |  |  |  |
| (Intercept) | 3.91 | 6.18 | 0.92 - 6.90 | **0.012** | 33.0 |
| time since infect | -0.08 | -0.01 | -0.24 - 0.08 | 0.310 | 33.0 |
| BW | 0.00 | -0.03 | -0.04 – 0.04 | 0.933 | 33.0 |
| R^2^ | 0.311 |  |  |  |  |

Table 5. Robust linear Regression. Influence of time since infection and the covariates on “General Stress” at t_1_.

|  | **General Stress** | | | | |
| --- | --- | --- | --- | --- | --- |
| *Predictors* | *Estimates* | *std. Beta* | *CI* | *p* | *df* |
| (Intercept) | 1.15 | 0.76 | 0.60 – 1.69 | **<0.001** | 95.00 |
| time since infect | 0.09 | 0.03 | -0.01 – 0.19 | 0.074 | 95.00 |
| R^2^ | 0.079 |  |  |  |  |
| (Intercept) | 1.50 | 0.81 | 0.64 – 2.36 | **0.001** | 94.00 |
| time since infect | 0.08 | 0.03 | -0.03 – 0.19 | 0.135 | 94.00 |
| Sex | -0.50 | -0.04 | -1.39 – 0.38 | 0.260 | 94.00 |
| R^2^ |  | 0.111 |  |  |  |
| (Intercept) | 0.88 | 0.32 | -0.25 – 2.00 | 0.464 | 94.00 |
| time since infect | 0.08 | 0.03 | -0.02 – 0.18 | 0.415 | 94.00 |
| age | 0.01 | 0.01 | -0.02 – 0.04 | 0.269 | 94.00 |
| R^2^ |  |  |  |  |  |
| (Intercept) |  |  | -1.72 – 4.07 | 0.424 | 91.00 |
| time since infect | 0.09 | 0.03 | -0.02 – 0.19 | 0.098 | 91.00 |
| BMI | 0.00 | -0.02 | -0.12 – 0.12 | 0.999 | 91.00 |
| R^2^ |  |  |  |  |  |
| (Intercept) | 1.64 | 0.86 | 0.10 – 3.18 | **0.037** | 94.00 |
| time since infect | 0.09 | 0.03 | -0.02 – 0.19 | 0.106 | 94.00 |
| LBM | -0.01 | -0.00 | -0.03 – 0.02 | 0.493 | 94.00 |
| R^2^ | 0.070 |  |  |  |  |
| (Intercept) | 2.29 | -0.09 | -12.37 – 16.94 | 0.753 | 33.00 |
| time since infect | 0.16 | 0.03 | -0.75 – 1.06 | 0.726 | 33.00 |
| BW | -0.02 | 0.01 | -0.22 – 0.18 | 0.852 | 33.00 |
| R^2^ | 0.159 |  |  |  |  |

Table 6. Robust linear Regression. Influence of time since infection and the covariates on “Emotional Stress” at t_1_.

|  | **Emotional Stress** | | | | | |  | | |  | | | |
| --- | --- | --- | --- | --- | --- | --- | --- | --- | --- | --- | --- | --- | --- |
| *Predictors* | *Estimates* | *std. Beta* | | *CI* | | *p* | | | *df* | | |  |  |
| (Intercept) | 1.45 | 1.69 | | 0.60 – 2.31 | | **0.001** | | | 34.00 | | | |  |
| time since infect | 0.07 | -0.13 | | -0.12 – 0.26 | | 0.441 | | | 34.00 | | | |  |
| R^2^ | 0.066 |  | |  | |  | | |  | | | |  |
| (Intercept) | 1.29 | 1.67 | | -0.37 –2.94 | | 0.123 | | | 33.00 | | | |  |
| time since infect | 0.07 | -0.13 | | -0.10 – 0.25 | | 0.412 | | | 33.00 | | | |  |
| age | 0.00 | 0.00 | | -0.04 – 0.05 | | 0.812 | | | 33.00 | | | |  |
| R^2^ | 0.069 |  | |  | |  | | |  | | | |  |
| (Intercept) | 1.42 | 1.57 | | 0.42 – 2.41 | | **0.007** | | | 33.00 | | | |  |
| time since infect | 0.07 | -0.12 | | -0.10 – 0.25 | | 0.416 | | | 33.00 | | | |  |
| Sex | 0.07 | 0.14 | | -0.93 – 1.06 | | 0.893 | | | 33.00 | | | |  |
| R^2^ | 0.067 |  | |  | |  | | |  | | | |  |
| (Intercept) | 1.25 | -0.13 | | -6.95 – 9.45 | | 0.758 | | | 33.00 | | | |  |
| time since infect | 0.07 | -0.18 | | -0.45 – 0.59 | | 0.788 | | | 33.00 | | | |  |
| BMI | 0.01 | 0.08 | | -0.35 – 0.36 | | 0.960 | | | 33.00 | | | |  |
| R^2^ | 0.067 |  | |  | |  | | |  | | | |  |
| (Intercept) | 01.20 | -0.54 | | -1.15 – 3.19 | | 0.345 | | | 27.00 | | | |  |
| time since infect | 0.07 | -0.01 | | -0.08 – 0.22 | | 0.334 | | | 27.00 | | | |  |
| LBM | 0.01 | 0.03 | | -0.03 – 0.04 | | 0.732 | | | 27.00 | | | |  |
| R^2^ | 0.086 |  | |  | |  | | |  | | | |  |
| (Intercept) | 1.42 | 1.57 | | 0.42 – 2.41 | | **0.007** | | | 33.00 | | | |  |
| time since infect | 0.07 | -0.12 | | -0.10 – 0.25 | | 0.416 | | | 33.00 | | | |  |
| BW | 0.07 | 0.14 | | -0.93 – 1.06 | | 0.893 | | | 33.00 | | | |  |
| R^2^ | 0.067 |  |  | |  | | |  | | |  |  |  |

Table 7. Robust linear Regression. Influence of time since infection and the covariates on “Social Stress” at t_1._

|  | **Social Stress** | | |  |  |
| --- | --- | --- | --- | --- | --- |
|  |  | | |  |  |
| *Predictors* | *Estimates* | *std. Beta* | *CI* | *p* | *df* |
| (Intercept) | 1.42 | 0.87 | 0.86 – 1.99 | **<0.001** | 34.00 |
| time since infect | 0.06 | 0.14 | -0.04 – 0.17 | 0.250 | 34.00 |
| R^2^ | 0.057 |  |  |  |  |
| (Intercept) | 1.57 | 0.41 | 0.01 – 3.13 | 0.049 | 33.00 |
| time since infect | 0.06 | 0.15 | -0.07 – 0.19 | 0.344 | 33.00 |
| Age | -0.00 | 0.01 | -0.04 – 0.04 | 0.832 | 33.00 |
| R2 | 0.060 |  |  |  |  |
| (Intercept) | 0.78 | 1.52 | -1.14 – 2.70 | 0.411 | 27.00 |
| time since infect | 0.06 | 0.15 | -0.08 – 0.19 | 0.393 | 27.00 |
| LBM | 0.01 | -0.01 | -0.02 – 0.04 | 0.564 | 27.00 |
| R^2^ | 0.087 |  |  |  |  |
| (Intercept) | 1.35 | 0.87 | 0.69 – 2.00 | **<0.001** | 33.00 |
| time since infect | 0.06 | 0.14 | -0.04 – 0.16 | 0.226 | 33.00 |
| Sex | 0.14 | 0.02 | -0.57 – 0.86 | 0.689 | 33.00 |
| R^2^ | 0.063 |  |  |  |  |
| (Intercept) | 0.81 | 1.15 | -1.20 – 2.82 | 0.419 | 33.00 |
| time since infect | 0.05 | 0.15 | -0.06 – 0.16 | 0.374 | 33.00 |
| BW | 0.01 | -0.00 | -0.02 – 0.04 | 0.522 | 33.00 |
| R^2^ | 0.075 |  |  |  |  |
| (Intercept) | 0.61 | 2.99 | -3.34 – 4.56 | 0.755 | 33.00 |
| time since infect | 0.05 | 0.16 | -0.10 – 0.20 | 0.511 | 33.00 |
| BMI | 0.04 | -0.10 | -0.14 – 0.21 | 0.677 | 33.00 |
| R2 | 0.074 |  |  |  |  |

Table 8. Robust linear Regression. Influence of time since infection and the covariates on “Performance Pressure” at t_1._

|  | **Performance Pressure** | | | |  |
| --- | --- | --- | --- | --- | --- |
| *Predictors* | *Estimates* | *std. Beta* | *CI* | *p* | *df* |
| Intercept) | 0.49 | 02.50 | 0.78 – 2.21 | <0.001 | 34.00 |
| time since infect | 0.10 | -0.10 | -0.06 – 0.26 | 0.229 | 34.00 |
| R^2^ | 0.109 |  |  |  |  |
| (Intercept) | 0.73 | 0.19 | -2.80 – 4.26 | 0.676 | 33.00 |
| time since infect | 0.09 | -0.17 | -0.15 – 0.32 | 0.460 | 33.00 |
| BMI | 0.03 | 0.09 | -0.12 – 0.19 | 0.664 | 33.00 |
| R^2^ | 0.120 |  |  |  |  |
| (Intercept) | 01.73 | 02.53 | 0.29 – 3.17 | 0.020 | 33.00 |
| time since infect | 0.10 | 0.15 | -0.02 – 0.22 | 0.108 | 33.00 |
| age | -0.01 | -0.04 | -0.04 – 0.03 | 0.708 | 33.00 |
| R^2^ | 0.115 |  |  |  |  |
| (Intercept) | 01.52 | 2.02 | 0.66 – 2.38 | 0.001 | 33.00 |
| time since infect | 0.10 | -0.10 | -0.06 – 0.25 | 0.206 | 33.00 |
| Sex | -0.05 | 0.12 | -0.89 – 0.79 | 0.902 | 33.00 |
| R^2^ | 0.110 |  |  |  |  |
| (Intercept) | 01.20 | 02.11 | -0.89 – 2.93 | 0.283 | 27.00 |
| time since infect | 0.10 | -0.05 | -0.09 – 0.28 | 0.298 | 27.00 |
| LBM | 0.01 | -0.01 | -0.03 – 0.04 | 0.683 | 27.00 |
| R^2^ | 0.134 |  |  |  |  |
| (Intercept) | 01. 10 | 1.00 | -1.44 – 3.64 | 0.385 | 33.00 |
| time since infect | 0.09 | -0.15 | -0.10 – 0.28 | 0.345 | 33.00 |
| bodyweight | 0.01 | 0.02 | -0.03 – 0.04 | 0.745 | 33.00 |
| R^2^ | 0.075 |  |  |  |  |

Table 9. Robust linear Regression. Influence of time since infection and the covariates on “Overtiredness” at t_1._

|  | **Overtiredness** | | | |  |
| --- | --- | --- | --- | --- | --- |
| *Predictors* | *Estimates* | *std. Beta* | *CI* | *p* | *df* |
| (Intercept) | 1.78 | 1.15 | 0.73 – 2.82 | 0.002 | 34.00 |
| time since infect | 0.14 | 0.17 | -0.05 – 0.33 | 0.142 | 34.00 |
| R^2^ | 0.139 |  |  |  |  |
| (Intercept) | 2.31 | 0.45 | -1.21 – 5.83 | 0.190 | 27.00 |
| time since infect | 0.12 | 0.15 | -0.12 – 0.36 | 0.310 | 27.00 |
| LBM | -0.01 | 0.01 | -0.07 – 0.05 | 0.780 | 27.00 |
| R^2^ | 0.107 |  |  |  |  |
| (Intercept) | 1..88 | 0.92 | 0.03 – 3.73 | 0.004 | 33.00 |
| time since infect | 0.14 | 0.16 | -0.12 – 0.41 | **<0.001** | 33.00 |
| Sex | -0.20 | 0.43 | -2.19 – 1.79 | 0.199 | 33.00 |
| R^2^ | 0.144 |  |  |  |  |
| (Intercept) | 1.31 | 1.42 | -1.18 – 3.80 | 0.291 | 33.00 |
| time since infect | 0.14 | 0.17 | -0.06 – 0.34 | 0.169 | 33.00 |
| age | 0.01 | -0.01 | -0.05 – 0.08 | 0.676 | 33.00 |
| R^2^ | 0.152 |  |  |  |  |
| (Intercept) | -0.50 | -1.82 | -5.25 – 4.25 | 0.832 | 33.00 |
| time since infect | 0.11 | 0.10 | -0.08 – 0.31 | 0.239 | 33.00 |
| BMI | 0.10 | 0.14 | -0.10 – 0.30 | 0.329 | 33.00 |
| R^2^ | 0.190 |  |  |  |  |
| (Intercept) | 0.94 | -2.89 | -1.56 – 3.43 | 0.451 | 33.00 |
| time since infect | 0.13 | 0.05 | 0.00 – 0.25 | **0.042** | 33.00 |
| BW | 0.01 | 0.06 | -0.02 – 0.05 | 0.479 | 33.00 |
| R^2^ | 0.151 |  |  |  |  |

Table 10. Robust linear Regression. Influence of time since infection and the covariates on “Lack of Energy” at t_1_.

|  | **Lack of Energy** | | | |  |
| --- | --- | --- | --- | --- | --- |
| *Predictors* | *Estimates* | *std. Beta* | *CI* | *p* | *df* |
| (Intercept) | 1.86 | 1.26 | 0.73 – 2.98 | 0.002 | 34.00 |
| time since infect | 0.11 | 0.05 | -0.10 – 0.32 | 0.282 | 34.00 |
| R^2^ | 0.091 |  |  |  |  |
| (Intercept) | 1.55 | 0.02 | -3.25-6.35 | 0.517 | 33.00 |
| time since infect | 0.11 | -0.00 | -0.17 – 0.38 | 0.437 | 33.00 |
| BW | 0.00 | 0.02 | -0.06-0.07 | 0.891 | 33.00 |
| R^2^ | 0.093 |  |  |  |  |
| (Intercept) | 1.99 | 0.54 | -2.30 – 6.28 | 0.350 | 27.00 |
| time since infect | 0.09 | 0.03 | -0.21 – 0.39 | 0.561 | 27.00 |
| LBM | -0.00 | 0.01 | -0.08 – 0.07 | 0.967 | 27.00 |
| R^2^ | 0.059 |  |  |  |  |
| (Intercept) | 01.11 | -0.05 | -4.88 – 7.10 | 0.709 | 33.00 |
| time since infect | 0.10 | 0.01 | -0.15 – 0.35 | 0.420 | 33.00 |
| BMI | 0.03 | 0.06 | -0.22 – 0.29 | 0.796 | 33.00 |
| R^2^ | 0.098 |  |  |  |  |
| (Intercept) | 01.20 | 01.87 | -1.13 – 3.53 | 0.302 | 33.00 |
| time since infect | 0.11 | 0.03 | -0.09 – 0.30 | 0.260 | 33.00 |
| age | 0.02 | -0.01 | -0.04 – 0.08 | 0.526 | 33.00 |
| R^2^ | 0.119 |  |  |  |  |
| (Intercept) | 01.87 |  | 0.64 – 3.10 | 0.004 | 33.00 |
| time since infect | 0.11 | 0.02 | -.07 – 0.29 | 0.222 | 33.00 |
| Sex | -0.02 | 0.35 | -1.36 – 1.32 | 0.978 | 33.00 |
| R^2^ | 0.091 |  |  |  |  |

Table 11. Robust linear Regression. Influence of time since infection and the covariates on “Somatic Stress” at t_1_.

|  | **Somatic Stress** |  | | |  |
| --- | --- | --- | --- | --- | --- |
| *Predictors* | *Estimates* | *std. Beta* | *CI* | *p* | *df* |
| (Intercept) | 01.42 | 0.76 | **0.48 – 2.37** | **0.004** | 34.00 |
| time since infect | 0.12 | 0.16 | -0.05 – 0.30 | 0.156 | 34.00 |
| R^2^ | 0.067 |  |  |  |  |
| (Intercept) | -0.68 | 0.93 | -5.46 – 4.10 | 0.774 | 33.00 |
| time since infect | 0.09 | 0.16 | -0.11 – 0.28 | 0.373 | 33.00 |
| BMI | 0.09 | -0.01 | -0.11 – 0.29 | 0.368 | 33.00 |
| R^2^ | 0.031 |  |  |  |  |
| (Intercept) | 01.78 | 02.61 | 0.31 – 3.25 | **0.019** | 33.00 |
| time since infect | 0.13 | 0.14 | -0.09 – 0.35 | 0.240 | 33.00 |
| Sex | -0.54 | -1.82 | -2.16 – 1.09 | 0.506 | 33.00 |
| R^2^ | 0.122 |  |  |  |  |
| (Intercept) | 01.60 | 01.39 | -2.20 – 5.40 | 0.399 | 33.00 |
| time since infect | 0.13 | 0.17 | -0.07 – 0.33 | 0.205 | 33.00 |
| BW | -0.00 | -0.01 | -0.05 – 0.05 | 0.935 | 33.00 |
| R^2^ | 0.074 |  |  |  |  |
| (Intercept) | 0.23 | -0.18 | -1.28 – 1.74 | 0.758 | 33.00 |
| time since infect | 0.11 | 0.13 | -0.02 – 0.24 | 0.085 | 33.00 |
| age | 0.03 | 0.04 | -0.00 – 0.07 | 0.083 | 33.00 |
| R^2^ | 0.096 |  |  |  |  |
| (Intercept) | 01.72 | 2.00 | -1.57 – 5.00 | **0.012** | 27.00 |
| time since infect | 0.12 | 0.18 | -0.10 – 0.34 | **<0.001** | 27.00 |
| LBM | -0.01 | -0.03 | -0.06 – 0.05 | 0.064 | 27.00 |
| R^2^ | 0.067 |  |  |  |  |

Table 12. Robust linear Regression. Influence of time since infection and the covariates on “Success” at t_1_.

|  | **Success** |  |  |  |  |
| --- | --- | --- | --- | --- | --- |
| *Predictors* | *Estimates* | *std. Beta* | *CI* | *p* | *df* |
| (Intercept) | 02.33 | 03.08 | 1.65 – 3.01 | **<0.001** | 34.00 |
| time since infect | 0.00 | -0.11 | -0.12 – 0.13 | 0.978 | 34.00 |
| R^2^ | -0.000 |  |  |  |  |
| (Intercept) | 0.91 | -1.16 | -3.19-5.00 | 0.656 | 33.00 |
| time since infect | -0.02 | -0.12 | -0.23-0.19 | 0.829 | 33.00 |
| BW | 0.02 | 0.06 | -0.04-0.08 | 0.487 | 33.00 |
| R^2^ | 0.077 |  |  |  |  |
| (Intercept) | 01.32 | 03.24 | -1.21 – 3.85 | 0.295 | 27.00 |
| time since infect | 0.02 | -0.07 | -0.16 – 0.20 | 0.841 | 27.00 |
| LBM | 0.02 | -0.01 | -0.03 – 0.06 | 0.470 | 27.00 |
| R^2^ | 0.065 |  |  |  |  |
| (Intercept) | 02.06 | 03.08 | 1.18 – 2.95 | **<0.001** | 33.00 |
| time since infect | 0.00 | -0.11 | -0.13 – 0.13 | 0.989 | 33.00 |
| Sex | 0.43 | -0.03 | -0.52 – 1.38 | 0.360 | 33.00 |
| R^2^ | 0.044 |  |  |  |  |
| (Intercept) | 02.49 | 02.88 | 1.02 – 3.96 | **0.002** | 33.00 |
| time since infect | 0.00 | -0.11 | -0.12 – 0.13 | 0.938 | 33.00 |
| age | -0.01 | 0.01 | -0.04 – 0.03 | 0.784 | 33.00 |
| R^2^ | 0.005 |  |  |  |  |
| (Intercept) | 01.02 | -0.16 | -3.26 – 5.30 | 0.631 | 33.00 |
| time since infect | -0.01 | -0.14 | -0.18 – 0.15 | 0.865 | 33.00 |
| BMI | 0.06 | 0.15 | -0.13 – 0.24 | 0.543 | 33.00 |
| R^2^ | 0.037 |  |  |  |  |

Table 13. Robust linear Regression. Influence of time since infection and the covariates on “Somatic Recovery” at t_1_.

|  | **Somatic Recovery** | | |  |  |
| --- | --- | --- | --- | --- | --- |
| *Predictors* | *Estimates* | *std. Beta* | *CI* | *p* | *df* |
| (Intercept) | 03.13 | 03.36 | 2.41 – 3.85 | **<0.001** | 34.00 |
| time since infect | -0.08 | -0.13 | -0.21 – 0.05 | 0.231 | 34.00 |
| R^2^ | 0.046 |  |  |  |  |
| (Intercept) | 03.95 | 04. 38 | 2.33 – 5.58 | **<0.001** | 33.00 |
| time since infect | -0.07 | -0.09 | -0.21 – 0.06 | 0.277 | 33.00 |
| age | -0.02 | -0.03 | -0.07 – 0.02 | 0.235 | 33.00 |
| R^2^ | 0.100 |  |  |  |  |
| (Intercept) | 02.78 | 01.79 | 0.79 – 4.78 | **0.008** | 27.00 |
| time since infect | -0.06 | -0.11 | -0.20 – 0.08 | 0.398 | 27.00 |
| LBM | 0.00 | 0.02 | -0.03 – 0.04 | 0.792 | 27.00 |
| R^2^ | 0.029 |  |  |  |  |
| (Intercept) | 02.87 | 03.34 | 1.96 – 3.78 | **<0.001** | 33.00 |
| time since infect | -0.08 | -0.13 | -0.21 – 0.06 | 0.240 | 33.00 |
| sex | 0.39 | 0.06 | -0.60 – 1.38 | 0.427 | 33.00 |
| R^2^ | 0.069 |  |  |  |  |
| (Intercept) | 04.73 | 07.06 | 0.55 – 8.90 | **0.028** | 33.00 |
| time since infect | -0.07 | 0.08 | -0.25 – 0.12 | 0.475 | 33.00 |
| BMI | -0.07 | -0.17 | -0.24 – 0.11 | 0.453 | 33.00 |
| R^2^ | 0.061 |  |  |  |  |
| (Intercept) | 03.21 | 05.64 | -0.49 – 6.91 | 0.087 | 33.00 |
| time since infect | -0.08 | 0.06 | -0.28 – 0.12 | 0.443 | 33.00 |
| BW | -0.00 | -0.04 | -0.05 – 0.05 | 0.958 | 33.00 |
| R^2^ | 0.046 |  |  |  |  |

Table 14. Robust linear Regression. Influence of time since infection and the covariates on “General Recovery” at t_1_.

|  | **General Recovery** | | |  |  |
| --- | --- | --- | --- | --- | --- |
| *Predictors* | *Estimates* | *std. Beta* | *CI* | *p* | *df* |
| (Intercept) | 03.70 | 03.71 | 3.06 – 4.34 | **<0.001** | 34.00 |
| time since infect | -0.06 | 0.03 | -0.18 – 0.06 | 0.326 | 34.00 |
| R^2^ | 0.038 |  |  |  |  |
| (Intercept) | 04.72 | 04.80 | 2.55 – 4.89 | **<0.001** | 33.00 |
| time since infect | -0.06 | -0.07 | -0.23 – 0.11 | 0.489 | 33.00 |
| Sex | -0.03 | -1.18 | -1.29 – 1.24 | 0.968 | 33.00 |
| R^2^ | 0.038 |  |  |  |  |
| (Intercept) | 04.77 | 08.30 | -0.46 – 10.00 | 0.073 | 33.00 |
| time since infect | -0.04 | 0.08 | -0.39 – 0.30 | 0.802 | 33.00 |
| BW | -0.01 | -0.06 | -0.08 – 0.06 | 0.710 | 33.00 |
| R^2^ | 0.001 |  |  |  |  |
| (Intercept) | 04.49 | 06.40 | 0.22 – 8.76 | **0.040** | 33.00 |
| time since infect | -0.05 | 0.04 | -0.22 – 0.13 | 0.581 | 33.00 |
| BMI | -0.03 | -0.16 | -0.21 – 0.15 | 0.704 | 33.00 |
| R^2^ | 0.049 |  |  |  |  |
| (Intercept) | 04.22 | 04.64 | 2.97 – 5.48 | **<0.001** | 33.00 |
| time since infect | -0.06 | -0.05 | -0.16 – 0.05 | 0.276 | 33.00 |
| age | -0.01 | -0.03 | -0.05 – 0.02 | 0.350 | 33.00 |
| R^2^ | 0.065 |  |  |  |  |
| (Intercept) | 03.62 | 06.22 | 0.77 – 6.48 | **0.015** | 27.00 |
| time since infect | -0.04 | -0.11 | -0.22 – 0.15 | 0.676 | 27.00 |
| LBM | -0.00 | -0.05 | -0.05 – 0.05 | 0.990 | 27.00 |
| R^2^ | 0.018 |  |  |  |  |

Table 15. Robust linear Regression. Influence of time since infection and the covariates on “Sleep” at t_1_.

|  | **Sleep** | | |  |  |
| --- | --- | --- | --- | --- | --- |
| *Predictors* | *Estimates* | *std. Beta* | *CI* | *p* | *df* |
| (Intercept) | 02.54 | 02.19 | 2.17 – 2.91 | **<0.001** | 34.00 |
| time since infect | -0.01 | 0.04 | -0.08 – 0.06 | 0.820 | 34.00 |
| R^2^ | 0.005 |  |  |  |  |
| (Intercept) | 02.20 | 01.73 | 0.90 – 3.49 | **0.002** | 33.00 |
| time since infect | -0.01 | 0.00 | -0.08 – 0.06 | 0.709 | 33.00 |
| bodyweight | 0.00 | 0.01 | -0.01 – 0.02 | 0.593 | 33.00 |
| R^2^ | 0.011 |  |  |  |  |
| (Intercept) | 02.06 | 02.55 | 0.07 – 4.04 | **0.043** | 33.00 |
| time since infect | -0.01 | 0.02 | -0.10 – 0.07 | 0.747 | 33.00 |
| BMI | 0.02 | -0.01 | -0.06 – 0.11 | 0.616 | 33.00 |
| R^2^ | -0.002 |  |  |  |  |
| (Intercept) | 02.55 | 02.19 | 2.11 – 2.99 | **<0.001** | 33.00 |
| time since infect | -0.01 | 0.00 | -0.07 – 0.06 | 0.836 | 33.00 |
| Sex | -0.02 | 0.24 | -0.50 – 0.45 | 0.922 | 33.00 |
| R^2^ | 0.002 |  |  |  |  |
| (Intercept) | 01.94 | 01.50 | 1.16 – 2.71 | **<0.001** | 33.00 |
| time since infect | -0.01 | 0.03 | -0.08 – 0.05 | 0.690 | 33.00 |
| age | 0.02 | 0.03 | -0.00 – 0.04 | 0.081 | 33.00 |
| R^2^ | 0.145 |  |  |  |  |
| (Intercept) | 02.93 | 01.94 | 1.70 – 4.17 | **<0.001** | 27.00 |
| time since infect | -0.01 | 0.01 | -0.09 – 0.08 | 0.861 | 27.00 |
| LBM | -0.01 | 0.01 | -0.03 – 0.02 | 0.567 | 27.00 |
| R^2^ | -0.003 |  |  |  |  |
